# Brain-optimized deep neural network models of human visual areas learn non-hierarchical representations

**DOI:** 10.1038/s41467-023-38674-4

**Published:** 2023-06-07

**Authors:** Ghislain St-Yves, Emily J. Allen, Yihan Wu, Kendrick Kay, Thomas Naselaris

**Affiliations:** 1 Department of Neuroscience, University of Minnesota, Minneapolis, MN 55455 USA; 2 Center for Magnetic Resonance Research, University of Minnesota, Minneapolis, MN 55455 USA; 3 Department of Psychology, University of Minnesota, Minneapolis, MN 55455 USA; 4 Graduate Program in Cognitive Science, University of Minnesota, Minneapolis, MN 55455 USA; 5 Department of Radiology, University of Minnesota, Minneapolis, MN 55455 USA

**Keywords:** Neural encoding, Pattern vision, Computational neuroscience

## Abstract

Deep neural networks (DNNs) optimized for visual tasks learn representations that align layer depth with the hierarchy of visual areas in the primate brain. One interpretation of this finding is that hierarchical representations are necessary to accurately predict brain activity in the primate visual system. To test this interpretation, we optimized DNNs to directly predict brain activity measured with fMRI in human visual areas V1-V4. We trained a single-branch DNN to predict activity in all four visual areas jointly, and a multi-branch DNN to predict each visual area independently. Although it was possible for the multi-branch DNN to learn hierarchical representations, only the single-branch DNN did so. This result shows that hierarchical representations are not necessary to accurately predict human brain activity in V1-V4, and that DNNs that encode brain-like visual representations may differ widely in their architecture, ranging from strict serial hierarchies to multiple independent branches.

## Abstract (web summary)

Whether or not deep neural networks require hierarchical representations to predict brain activity is not known. Here, the authors show that a multi-branch deep neural network can predict neural activity independently in visual areas in the absence of hierarchical representations.

## Introduction

Deep neural networks (DNN) that have been trained to solve computer vision problems learn representations that can accurately predict the human brain’s response to complex real-world visual stimuli (e.g. photographs). There has been a considerable effort to understand this success. One hypothesis is that the predictive accuracy of DNNs depends, at least in part, upon their implementing a hierarchy of visual processing stages. This hypothesis is plausible because hierarchical processing is believed to be an important organizing principle of primate vision^1–5^; indeed, the architecture of some convolutional DNNs was inspired by the evidence for hierarchical processing in the primate visual system^6–8^. The hypothesis also has some experimental support from the finding that layer depth in task-optimized DNNs aligns to the hierarchical progression of distinct visual maps in primate cortex^9–14^. However, the possibility that non-hierarchical models might predict brain activity as accurately as models based on hierarchical, task-optimized DNNs has not been thoroughly investigated.

Here, we test if hierarchical representations are essential for encoding models that predict brain activity in response to visual stimuli with state-of-the-art (SOTA) accuracy. In performing this test, we acknowledge that “hierarchy” is a somewhat loaded term that has multiple, context-dependent meanings. Thus, we introduce and distinguish between three conceptually distinct kinds of hierarchy.

**Fig. 1:**
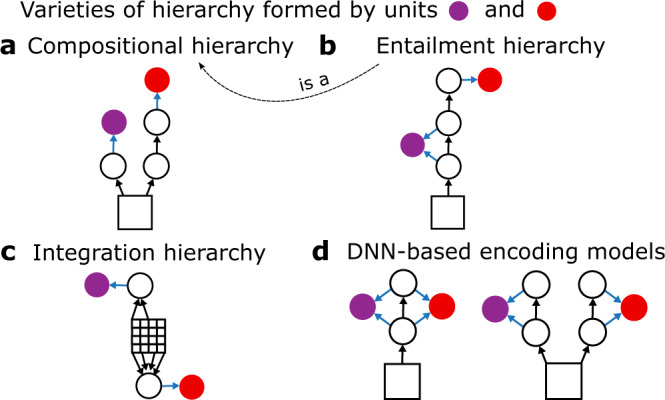
Varieties of hierarchy. Illustrations of different kinds of hierarchy formed by units of interest (filled circles) that perform linear read-out (blue arrows) of activity in a deep neural network (DNN; empty circles are units with nonlinear activation functions, black arrows are connections, squares are visual input). **a** In a *compositional hierarchy*, the red unit requires more compositions of the nonlinear activation function than the purple unit. **b** In an *entailment hierarchy*, all components of the network that activate the purple unit contribute to activation of the red unit, but not the other way around. The entailment hierarchy shown here is a compositional hierarchy (dashed arrow). The compositional hierarchy shown in (**a**) is not an entailment hierarchy unless the two branches happen to have functionally identical first layers. **c** In an *integration hierarchy*, the read-out connections of the red unit offer a wider spatial integration window (grid marks indicate pixels) than the purple unit. As shown, an integration hierarchy need not be a compositional or entailment hierarchy. **d** Connectivity diagrams illustrating encoding models with single-branch (left) and multi-branch (right) DNNs, where the units of interest are located in the brain. Under each model, the units of interest could form all three kinds of hierarchy defined in (**a**–**c**), or none, depending upon the specific values of the network and read-out connections.

In a *compositional hierarchy* higher-level representations require more nonlinear processing steps to compute than lower-level representations (Fig. 1a). This kind of hierarchy has been offered as an explanation for the “gradient of complexity” of representations in the primate visual system^11,15,16^.

In an *entailment hierarchy* low-level representations act as necessary pre-processing stages for higher-level representations (Fig. 1b). We refer to this as “entailment” because it implies that a successful DNN model of a higher brain area would have to include layers that successfully model lower brain areas. Although the relationship between anatomical and processing hierarchies is complex and not fully understood^17^, apparent support for entailment hierarchy comes from the finding that inactivation of primary visual cortex greatly reduces activity in V2^18^, V3^19^, and V4^20^.

In an *integration hierarchy*, the spatial integration windows (i.e., receptive field size) that underlie some representations are larger than others (Fig. 1c). The expansion of receptive field sizes with progression from lower to higher visual areas is arguably the most salient kind of hierarchy in the visual system, and has been demonstrated in many previous studies^21,22^.

These three kinds of hierarchy are conceptually and logically distinct, and it is relatively easy to specify networks that form one kind of hierarchy, but not others. The filled-in units in the network in Fig. 1a, for example, form a compositional hierarchy but not an entailment hierarchy (unless both branches happen to have functionally identical first layers). In the simple network shown in Fig. 1c the two filled-in units form an integration hierarchy because they pool over differently-sized regions of the input image, but do not form a compositional or entailment hierarchy because they process the input image independently using the same number of nonlinear function compositions (just one). Given that these kinds of hierarchy are not identical, it is possible that only some of the three kinds of hierarchy are essential for accurately predicting brain activity. It is also possible that none of them are necessary.

Although a purely feedforward DNN (e.g., AlexNet) most naturally embodies all three kinds of hierarchy, each kind of hierarchy could be formed by units in DNNs with a wide variety of different architectures, including DNNs with recurrent connections, skip connections, or multiple, independent branches. We reasoned that if a specific kind of hierarchy is important for accurately predicting brain activity, then any accurate DNN-based encoding model would show evidence for it, as long as the network and read-out connections admit at least one hierarchical solution. We therefore tested for hierarchy in three very distinct network-based encoding models of human visual areas V1–V4. Each encoding model consisted of a DNN coupled to a read-out head that transformed activity in the DNN into predictions of brain activity measured in individual voxels. For each voxel, read-out heads were permitted to sample from all layers of the DNN, thus giving encoding models the flexibility to learn representations that formed whatever kind of hierarchy was needed to most accurately predict brain activity. As in previous work^23^, in one of the models the DNN was a task-optimized AlexNet^7^ that was pre-trained to classify objects in the ImageNet database^24^. In the other two encoding models, the DNNs were directly optimized to predict human brain activity^25–33^ using a massive sampling of BOLD responses to hundreds of thousands of presentations of natural scenes^34^. Importantly, we constructed two different encoding models based on brain-optimized DNNs with very different architectures: a single-branch DNN that was trained to predict activity in all four visual areas jointly (Fig. 1d left, Fig. 4a), and a multi-branch DNN in which each branch was trained independently to predict a single visual area (Fig. 1d right, Fig. 4b). We then investigated the task- and brain-optimized encoding models to determine the kinds of hierarchy essential for predicting human brain activity in functionally diverse visual areas.

## Results

### Encoding models based on brain-optimized networks yield accurate predictions of brain activity to natural and artificial stimuli

**Fig. 2:**
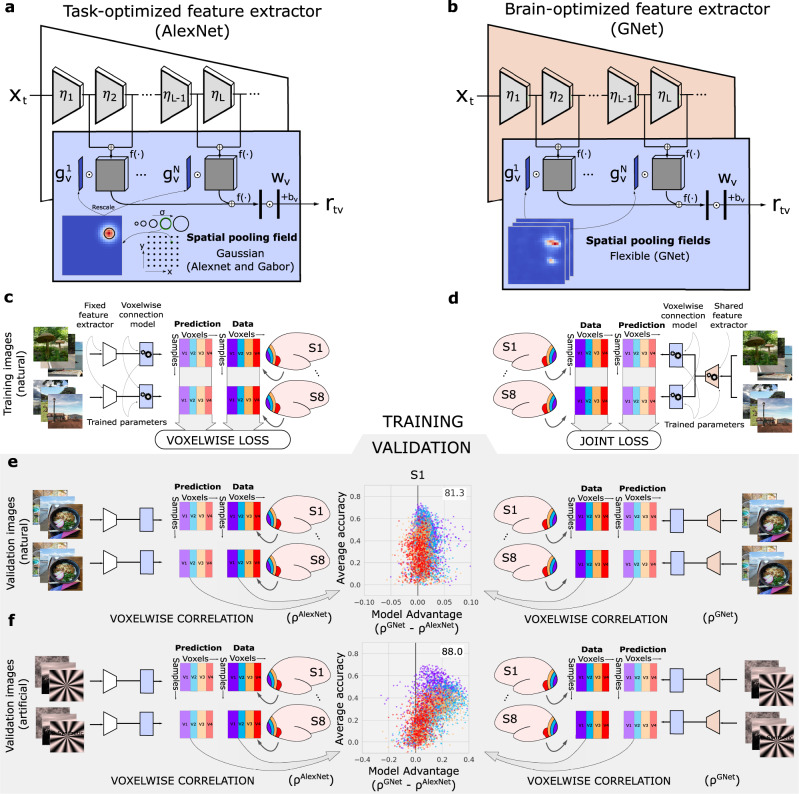
Training and validation of task-optimized and brain-optimized networks. **a** An encoding model based on a task-optimized deep neural network (AlexNet; white trapezoid). Multiple convolutional layers (*η*_*i*_) convert the input stimulus on trial *t*, *X*_*t*_, into feature maps. A read-out head (blue rectangle) transforms network activations into predicted brain activity (*r*_*tv*_, where *v* indexes a single voxel). The read-out head consists of Gaussian spatial pooling fields (${g}_{v}^{i}$; example at lower left) with position and size selected from a fixed grid of candidates (lower right). The pooling field and each feature map are multiplied pixel-wise and then summed, reducing each feature map to a single feature value. The array of feature values across all maps (left vertical rectangle) are weighted by an array of feature weights (*w*_*v*_) and then summed (with a bias term *b*_*v*_) to yield predicted brain activity. Compressive point nonlinearities (*f*( ⋅ )) are applied at several processing stages. **b** A similar architecture is used for the encoding model based on the brain-optimized network (GNet; orange trapezoid depicts a single-branch architecture). The “flexible” spatial pooling fields used in the read-out head may be non-Gaussian (example at lower left). **c** Training for the AlexNet-based encoding model. For each voxel, only read-out head parameters (*g*_*v*_, *w*_*v*_, *b*_*v*_) are optimized (gears) for visual areas V1–V4 (colored rectangles) in each subjects' brain (S1, …, S8). The “voxelwise loss” (squared difference between predictions and measured activity data) is evaluated independently for each voxel. **d** Training for the GNet-based encoding model. Both the read-out head and GNet parameters are optimized jointly ("joint loss'') for all voxels, subjects and brain areas. **e** Prediction accuracy is evaluated for each voxel by correlating predicted brain activity with measured brain activity (*ρ*^AlexNet^, *ρ*^GNet^) across a set of held-out validation trials with natural scenes. We plot the average accuracy (*y*-axis of central plot) vs. the difference in accuracy (*x*-axis) for each voxel (dots; color indicates visual area). For this example subject (S1) the GNet-based encoding model predicts responses to natural scenes most accurately for 81.3% of voxels in V1–V4. **f** In this example, the GNet-based encoding models predicts responses to artificial stimuli more accurately for 88% of voxels in V1–V4.

In encoding models based upon deep neural networks, the DNN acts as a nonlinear feature extractor, and the activities of units in each feature map of the DNN are transformed into predicted brain activity via a near-linear read-out head (Fig. 2; see St-Yves and Naselaris^23^ as well as Allen et al.^34^). For each voxel in the target dataset, the read-out head specifies a spatial receptive field and an array of feature weights that model the region of visual space and the nonlinear features that are represented by brain activity measured in the voxel. In our work the read-out head for each voxel always samples from layers throughout the depth of the network. Thus, we give each layer in the feature-extractor network the chance to contribute to predicting brain activity (Fig. 2a, b).

We constructed encoding models in which the feature extractor is a brain-optimized neural network (GNet, Fig. 2b). In the GNet encoding models, we minimized error on predicted brain activity by using stochastic gradient descent to learn all free parameters of the DNN feature extractor simultaneously with the free parameters of the read-out heads (Fig. 2d). We compared the prediction accuracy of these GNet-based encoding models to models based upon a task-optimized neural network (AlexNet^7^;) that was pre-trained to discriminate object categories. For the AlexNet encoding model (Fig. 2a) the network parameters were not optimized to predict brain activity; only the free parameters of the read-out heads were optimized to predict brain activity (Fig. 2c).

To optimize the parameters of each type of encoding model (Fig. 2c, d) we used the Natural Scenes Dataset^34^, a massive sampling of blood-oxygenation-level-depedendent (BOLD) activity in eight subjects using ultra-high field fMRI (7T, 1.8-mm resolution). Subjects each viewed 9000–10,000 natural scenes (sampled from the Microsoft Common Objects in Context database^35^) presented (3-s exposure) repeatedly (three times typically), yielding 22–30K trials for individual subjects and a total of 213K trials across subjects. Voxels were assigned to areas V1–V4 on the basis of an independent retinotopic mapping experiment^34^.

**Fig. 3:**
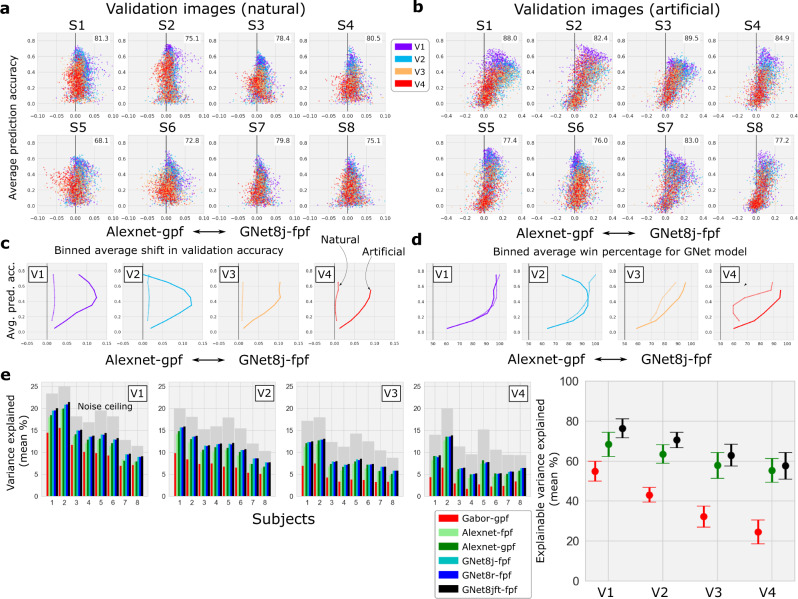
Comparison of cross-validated prediction accuracy for encoding models based on task- and brain-optimized deep neural networks. **a**, **b** Accuracy / advantage plots for all subjects and brain areas for natural validation stimuli (a) and artificial stimuli (b). Format as in Fig. 1. **c** Average difference in model prediction accuracy (*x*-axis) for different levels of average prediction accuracy (*y*-axis) and for natural (thin curve) and artificial (thick curve) stimuli. **d** Average percentage of voxels for which the GNet-based encoding model explains more signal variance than AlexNet-based encoding model ("win percentage''; x-axis) for different levels of average prediction accuracy (*y*-axis). **e** Signal variance (%; *y* axes) in brain activity explained by varieties of network-based encoding models (colored bars; gray bars indicate theoretical upper limit) for all subjects (x-axis) in visual areas V1–V4. Inset: Average (across subjects) percent of explainable variance explained by a subset of the models. See Supplementary Table 1 for descriptions of all model acronyms. Source data are provided as a Source Data file.

To validate and compare encoding models, after training we assessed the prediction accuracy of the models for each voxel by correlating predicted activity with measured activity in response to the shared images that were shown to all eight subjects during the experiment but were not used for model training (Fig. 2e). For each subject, the brain-optimized GNet encoding model (single-branch trained jointly on all brain areas) predicted brain activity in V1–V4 more accurately than the AlexNet encoding model for more than 68% of all voxels in V1–V4 (Fig. 3a). Averaged across subjects, the “win percentage” for the GNet model was significantly greater than expected by chance (80% win, *p* < 10^−6^, two-sided t-test). Interestingly, when model prediction accuracy was computed for out-of-sample classes of artificial stimuli (e.g., gratings, contrast-modulated scenes, various types of noise, Fig. 2f), the prediction accuracy of the GNet model was greater than the AlexNet model for more than 76% of the voxels in all subjects (Fig. 3b). Although the size of the difference in prediction accuracy underlying these win percentages varied within and across areas (Fig. 3c), across subjects the average win percentage of the GNet model for artificial stimuli was significantly greater than for natural stimuli (80% win, *p* < 10^−5^, two-sided *t* test). The win percentage was not significantly different across these two stimulus conditions for any single brain area except V4, where we observed an average improvement across subjects from 62% to 74% (*p* < 0.01, two-sided paired *t* test) (Fig. 3d).

These results demonstrate that the GNet model outperforms the AlexNet model for a majority of voxels in V1–V4. We also quantified the prediction accuracy of multiple model types with respect to the best performance that any model might achieve. To do this, we estimated the percentage of variance in brain activity that can be explained by variation in the stimulus (by computing noise ceilings as in Allen et al.^34^, gray bars in Fig. 3e), as well as the percentage of variance explained by the outputs of each model (colored bars in Fig. 3e). For every subject and brain area (with the exception of S4, V4), the mean (across all voxels) percentage of total variance explained by GNet models is larger than or equal to the mean percentage of variance explained by the AlexNet models and a control Gabor wavelet model. Improvements in prediction accuracy achieved by extensive fine-tuning of the model parameters (see Methods) suggests that the limit of prediction accuracy for GNet models has not yet been reached ("GNet8jft-fpf”, Fig. 3e, right). However, across all subjects and brain areas, GNet encoding models can account for at most 78% of the explainable variance, and as little as 37% (on average over a visual area, for voxels with at least 5% of explainable variance). This means that currently, even the best models leave much to be explained. Interestingly, this limitation does not seem to be unique to models trained on BOLD activity. For most subjects, the amount of explainable variance in V1 that is explained by GNet is similar to the amount explained by similar brain-optimized DNN-based models of single-unit activity in V1 of the primate brain^26^.

We conclude that our encoding models based upon brain-optimized networks are as trustworthy a tool for testing hypotheses about representations encoded in human brain activity as any currently existing network-based encoding model.

### Single- and multi-branch brain-optimized networks yield similar prediction accuracy

**Fig. 4:**
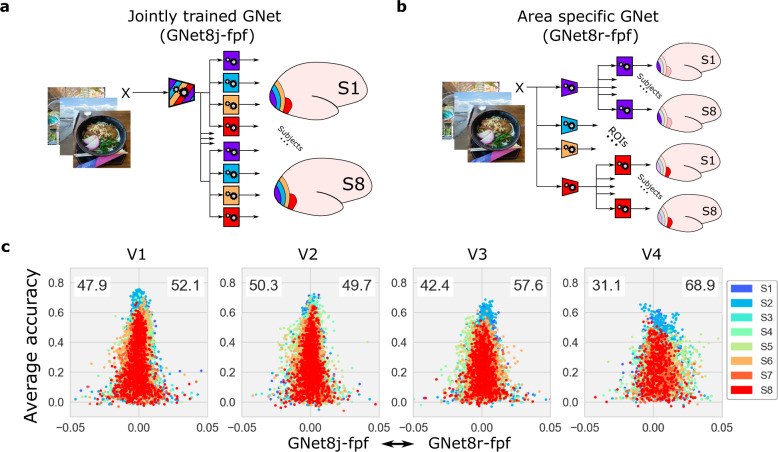
Comparison of brain-optimized DNNs trained jointly or independently on activity in V1–V4. **a** Training and architecture for the single-branch, jointly-trained (GNet8j-fpf) variant of the GNet-based encoding model. **b** A multi-branch variant (GNet8r-fpf). The feature extractor (trapezoids) in each branch is trained (gears) independently to predict a single brain area. In both cases, read-out heads (squares) with flexible pooling fields (fpf) are optimized as well. **c** Advantage/accuracy plots comparing prediction accuracy of encoding models based on the jointly-trained (left of vertical line in each panel) and independently-trained (right of vertical line) GNets for each subject (colors).

Like AlexNet, the GNet encoding model described above is a DNN with a single branch (Fig. 4a). We tested if comparable prediction accuracy could be obtained with an encoding model based upon a multi-branch architecture, in which the DNN feature extractor and and read-out head for each branch was trained independently of the other branches to predict activity in a specific visual area (Fig. 4b). Like the single-branch DNN model, this multi-branch variant can in principle learn encoding models that express all three kinds of hierarchy. We reasoned that if any kind of hierarchy was important for predicting brain activity, the DNN based upon the multi-branch DNN would express it or, if it did not, would yield relatively poor prediction accuracy. To distinguish these different brain-optimized encoding models we hereafter refer to the single-branch model as GNet8j, where “8” indicates the number of subjects that contributed training data, and “j” stands for “jointly trained”, and the multi-branch model as GNet8r, where “r” stands for “ROI-wise training”.

We found that GNet8j and GNet8r yielded nearly identical prediction accuracy. The win percentages for GNet8j and GNet8r were close to parity in V1–V3, although in V4 win percentage for GNet8r was 68%. Across several model variations, the win percentage of the GNet8j model was at best equal to the win percentage for the GNet8r model. This indicates that the jointly-trained, single-branch GNet model was not more accurate than the independently-trained branches of the GNet8r model (Fig. 4c). Thus, although GNet8r and GNet8j have very different architecture, they are equally plausible models of *representation* in V1–V4.

We note that the GNet8r model has many more tunable network parameters than GNet8j, and this difference in model capacity could, in principle, explain why the performance of GNet8r is on par with that of GNet8j. However, control analyses in which we equalize the capacity of different models revealed no clear advantage of one model class over the other across V1–V4 (Fig. S1).

### Single- and multi-branch brain-optimized networks both reveal hierarchical expansion of receptive field sizes

**Fig. 5:**
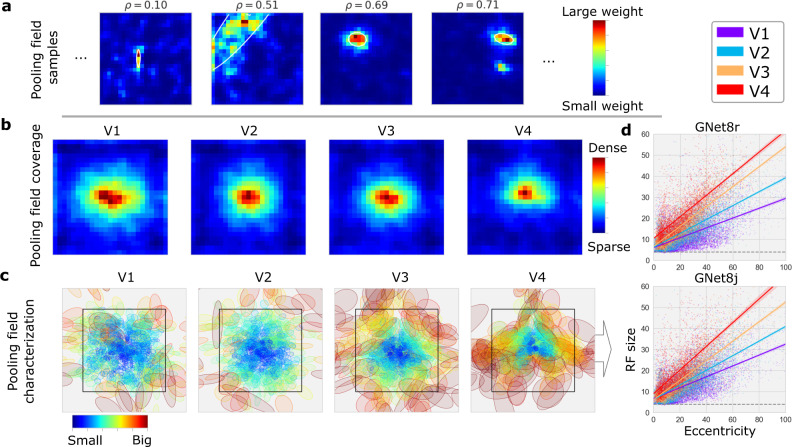
Encoding models based on brain-optimized networks recover known retinotopic organization. **a** Examples of spatial pooling fields (GNet8j-fpf model; *ρ* indicates prediction accuracy; colormap indicates strength of predicted activation) and best-fitting Gaussian profile (ellipsoids) for several individual voxels. **b** Mean of spatial pooling fields for all subjects and all voxels in V1–V4 with significantly accurate (*ρ* > 0.055, *p* < 0.01, permutation test) GNet8j encoding models. **c** Best-fitting Gaussian profiles for spatial pooling fields (same voxels as in (**b**)). These profiles were used to visualize size-eccentricity relationships for all visual areas. **d** Linear fits to relationship between pooling field size ($\sqrt{{{{{{\rm{area}}}}}}}$ of the one std. dev. elliptical Gaussian profile in (**c**)) and eccentricity for models GNet8r (top) and GNet8j (bottom). Length units are expressed in percent of stimulus span (i.e. 100% ≡ 8.4^∘^, black bounding box in (**c**)).

To test for evidence of integration hierarchy in both the single- and multi-branch GNet models, we analyzed the pooling fields learned by each model for each voxel in V1–V4. (Fig. 5a). Analysis of pooling revealed expected visual field coverage (Fig. 5b). Both models exhibited size-eccentricity relationships (Fig. 5c, d^22^) that fan out across areas, revealing a clear expansion of pooling field size with progression from V1–V4. Thus, as expected, both GNet-based encoding models learn representations that form an integration hierarchy.

### Single-branch task- and brain-optimized models learn compositional hierarchies; multi-branch brain-optimized models do not

Having established that single- and multi-branch DNN-based models had similar prediction accuracy and receptive field size-eccentricity relationships, we next tested if either showed evidence for compositional hierarchy.

**Fig. 6:**
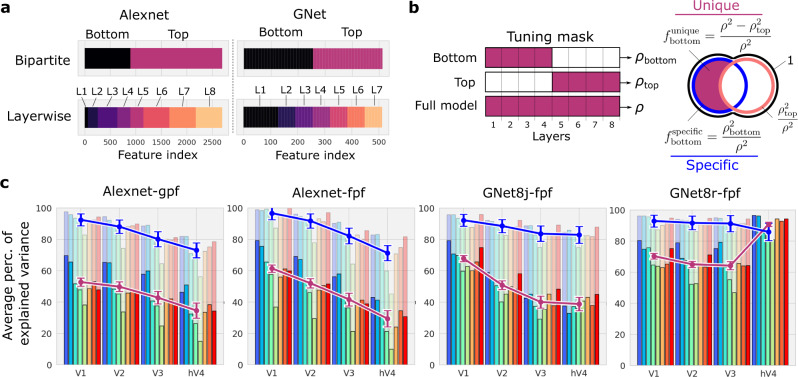
Alignment between layers and brain areas in task-optimized and brain-optimized networks. **a** Partitioning of layers (L1, L2,…) into bottom and top halves for AlexNet and GNet. **b** Partitioning of model output variance for bottom and top halves of the networks. Model predictions are generated using either the bottom or top layers alone, and the prediction accuracy is calculated (*ρ*_bottom_, *ρ*_top_). These quantities are used to calculate the “specific” (${f}_{{{{{{\rm{bottom}}}}}}}^{{{{{{\rm{specific}}}}}}}$) and “unique” (${f}_{{{{{{\rm{bottom}}}}}}}^{{{{{{\rm{unique}}}}}}}$) fractions of variance explained by the bottom layers. Both measure the contribution of the bottom layers to predicting activity in each brain area. **c** Specific (light bars, blue curve) and unique variance (solid bars, magenta curve) explained by the bottom layers for each brain area (x-axis) and all 8 subjects (colored bars) for variants of the AlexNet and GNet-based encoding models. The mean and error are estimated by sampling with replacement for all estimates of voxelwise validation accuracy and the error displayed is obtained via error propagation. Source data are provided as a Source Data File.

The read-out heads of the encoding model sample all layers of the underlying DNN branch. This allows all layers to compete to explain variance in the activity of each individual voxel. In a compositional hierarchy, we expect that read-out head connections that attach to lower layers of the DNN branch would be most important to explaining brain activity in V1, but increasingly less important with progression toward V4. Thus, we measured the contribution of layers in the bottom half of the feature-extracting DNNs to explaining variance in brain activity (Fig. 6, bold curves). We define the “specific” contribution of any set of layers as the prediction accuracy obtained when generating predictions with that set of layers alone (i.e., by masking out all other layers; Fig. 6b, c, blue curves). We define the “unique” contribution of any set of layers as the prediction accuracy of the full model, minus the specific contribution of all other layers (Fig. 6c, magenta curves). For two variants of the AlexNet encoding models ("AlexNet-gpf” and “AlexNet-fpf”), and for the multi-branch GNet encoding model ("GNet8j-fpf”), the unique contribution of lower layers declined monotonically from V1–V4 from roughly 60% to roughly 30%. Concurrently, the unique variance for the top layers (indicated by the distance from the top of the y-axis to the blue curves in Fig. 6c) increased monotonically from V1–V4. Thus, each of single-branch models seems to form a compositional hierarchy, in the sense that layer depth in these networks is aligned with the expected ordering of brain areas V1–V4. This finding is consistent with previous findings, most notably^11^.

In contrast, for the multi-branch model ("GNet8r-fpf”) there was little decline in the unique or specific contributions of the bottom layers from V1–V4. For V1–V3 the unique contribution of the lower layers was roughly 75%, and for V4 the unique contribution of the lower layers was above 90%. Thus, collectively the branches of the GNet8r model do not form a compositional hierarchy, as it is possible to express representations in V1–V4 with the same number of DNN layers. It is important to note that the jointly and independently trained GNet models utilized the same non-linearities, yield very similar prediction accuracy (Fig. 4c), and show similar RF expansions across visual areas. We conclude that compositional hierarchy is not needed to accurately predict brain activity.

### Single-branch task- and brain-optimized models learn entailment hierarchies; multi-branch brain-optimized models do not

We developed a test for entailment based upon transfer learning^36^. We applied the analysis to all model variants, starting with the AlexNet-based encoding model, as we expected it to show clear evidence for an entailment hierarchy. The intuition for this test is as follows: if representations in V1 are needed to express representations in V4, then a model that permits accurate read-out of V4 activity should permit accurate read-out of V1 activity as well; but a model that permits accurate read-out of V1 activity should not generalize to V4. In order to perform the transfer learning analysis, we first segregate the representations that predict each of the brain areas into distinct networks, and then test each network’s ability to cross-predict activity in other brain areas. In the case of the multi-branch GNet8r model, this segregation is built-in to the training. However, for the single-branch models, the segregation requires us to take the additional step of writing representations for each individual brain area into distinct networks.

**Fig. 7:**
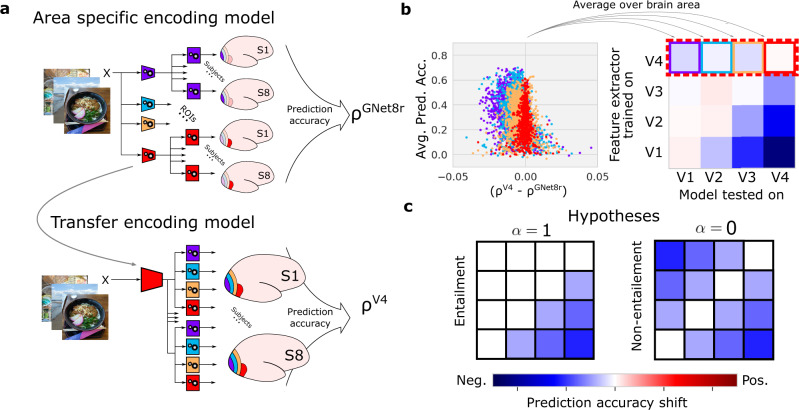
Testing entailment of representations using transfer learning. **a** Transfer learning procedure. Stand-alone GNet models consisting of a feature extractor (trapezoids) and a read-out head (squares) are trained (gears) to predict brain activity (shown), or the outputs of another encoding model (not shown) for V1–V4 independently. When trained on brain activity (as shown) the prediction accuracy of these “reference” models is just *ρ*^GNet8r^. The GNet feature extractors of these reference models are used to construct transfer models. For each specific brain area (V4 in this example) the GNet is frozen, and a new read-out head is trained for all areas, yielding prediction accuracy ${\rho }^{{V}_{j}}$, where the superscript indicates the brain area used to train the feature extractor. In this example *j* = 4 corresponding to area V4. **b** To determine how well representations transfer across brain areas, we compute the prediction accuracy shift (${\rho }_{v}^{{V}_{j}}-{\rho }_{v}^{{{{{{\rm{GNet8r}}}}}}}$, illustrated using an advantage/accuracy plot) for each voxel *v*. For each pair of brain areas *V*_*j*_, *V*_*i*_ we average these shifts over all voxels *v* ∈ *V*_*i*_, constructing a prediction accuracy shift matrix (at right is a hypothetical example). Rows of the matrix index the brain area used to train the feature extractor (*V*_*j*_); columns of the matrix index the brain area over which prediction accuracy shifts are averaged (*V*_*i*_). **c** For representations with entailment (*α* = 1), negative prediction accuracy shifts (blue) accumulate only below the diagonal of the matrix. For representations without entailment (*α* = 0) negative prediction accuracy shifts accumulate above and below the diagonal.

The transfer learning analysis began by first training four independent GNet models (i.e., a GNet feature extractor plus a read-out head) to predict the outputs (i.e., predicted brain activity) of the AlexNet model for V1, V2, V3, and V4, respectively. In other words, we treated the outputs of the AlexNet model as if they were synthetic brain data, and used the GNet8r modeling approach to copy its representations into four distinct branches of a multi-branch network (Fig. 7). The correlation between the outputs of these “reference” GNet models and the outputs of the original AlexNet model was near 1.0, indicating that we were able to (almost) losslessly copy the AlexNet model representations for each brain area into its own, separate branch. We denote this correlation ${\rho }_{v}^{{{{{{\rm{ref}}}}}}}$, where the subscript indexes voxels. If *v* ∈ *V*_*i*_, this should be read as the “prediction accuracy of a model with a GNet feature extractor and a read-out head trained and tested on *V*_*i*_”.

Next, we tested if the representations in each branch of the reference GNet model were transferable across branches. For each pair of brain areas (*V*_*i*_, *V*_*j*_), we froze the feature extractor of the *V*_*j*_ branch, and then trained a new read-out head to predict the outputs of the *V*_*i*_ branch. We then calculated the prediction accuracy of this new “transfer” model of *V*_*i*_. We denote this prediction accuracy ${\rho }_{v\in {V}_{i}}^{{V}_{j}}$. This should be read as the “prediction accuracy of a model with GNet feature extractor trained on *V*_*j*_, and read-out head trained and tested on a voxel *v* in *V*_*i*_” (Fig. 7a).

Finally, we constructed a 4 × 4 matrix of differences between the accuracy of reference and transfer models, averaged over all voxels (Fig. 7b). For each element (*i*, *j*) of the matrix we calculated:

$${\Delta }_{i}^{j}\equiv {\langle {\rho }_{v}^{{V}_{j}}-{\rho }_{v}^{{{{{{\rm{ref}}}}}}}\rangle }_{v\in {V}_{i}}$$

This “prediction accuracy shift matrix” reveals how accurately a DNN that is trained to model the representations in one brain area can model the representations in another brain area. In an entailment hierarchy, the representations in networks trained to model a more posterior area should be a subset of the representations in a network trained to model a more anterior area, but not vice versa. Thus, if strict entailment holds, all diagonal and upper-diagonal elements of this matrix should be zero, while all lower-diagonal elements should be negative (Fig. 7c).

**Fig. 8:**
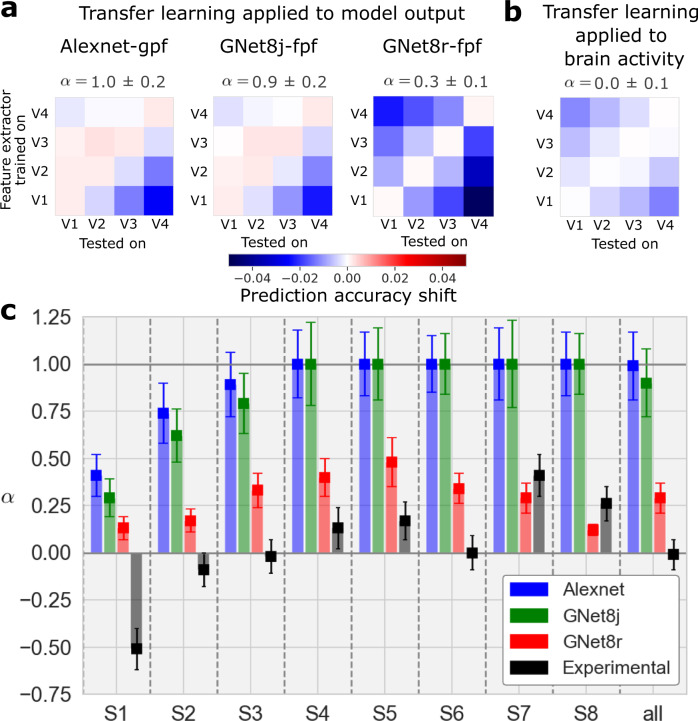
Transfer learning analysis for DNN-based encoding models and V1–V4. **a** Transfer learning applied to model outputs. Prediction accuracy shift matrices resulting from the transfer learning procedure applied to outputs of the AlexNet-gpf model, the GNet8j-fpf model, and the GNet8r-fpf model. **b** Transfer learning applied to brain activity. All matrices are averaged across 8 subjects. **c** Values of *α* estimated for individual subjects for the four test cases shown above. The error bars indicate the mean and SEM which are estimated from the matrix fitting procedure described in Methods. The shaded bar is added for emphasis. Source data are provided as a Source Data file.

As expected, the prediction accuracy shift matrix for the AlexNet model (Alexnet-gpf) closely resembled the ideal of strict entailment (Fig. 8a). An index we devised to measure resemblance to this ideal had a value of *α* = 1.0 ± 0.2, where *α* = 1 indicates strict entailment, and *α* = 0 indicates no entailment (and *α* = − 1 would indicate “reverse” entailment).

We conducted an identical analysis on the outputs of the single-branch GNet model (GNet8j-fpf) because, like the AlexNet-based encoding model, it exhibits all other forms of hierarchy. Again, and as expected, the prediction accuracy shift matrix closely resembled the ideal for strict entailment, with *α* = 0.9 ± 0.2.

In contrast, when this transfer learning analysis was applied to the outputs of the multi-branch GNet model (GNet8r-fpf), the prediction accuracy shift matrix most closely resembled the ideal outcome for non-entailment (*α* = 0.3 ± 0.1). Thus, we established that the GNet8r model learned representations that did not form an entailment hierarchy. We thus conclude the entailment hierarchy is not essential for accurately predicting brain activity.

Out of curiosity, we applied the transfer learning analysis to the measured brain activity itself (Fig. 8b, c, black bars). In this case, *ρ*^ref^ = *ρ*^GNet8r^ (as illustrated in Fig. 7). To calculate prediction accuracy shifts, we took the branch of GNet8r that was optimized to predict V4 brain activity (for instance), froze its parameters, and then re-trained read-out heads to predict brain activity in all areas (Fig. 7). We performed this transfer-learning procedure for feature extractors trained on every brain area. Interestingly, the resulting prediction accuracy shift matrix was most consistent with a lack of any entailment hierarchy (*α* = 0.0 ± 0.1). It is important to note, however, that when applying transfer learning directly to measured brain activity (as opposed to the outputs of other models), noise can impact the results in ways that make interpretation difficult.

### Outputs of models with hierarchical and non-hierarchical representations are strongly correlated

The differences in hierarchical representation between any pair of models implies that there must be differences between the models’ outputs (i.e., the activity predicted by each model in response to stimuli). To determine how great these difference were, we calculated the correlation of the outputs of each pair of models in response to the same set of 1000 images for each voxel.

When comparing either GNet8r or GNet8j to AlexNet, median correlation in model output decreased with progression toward higher areas. In all areas the correlation coefficients were widely distributed. This is consistent with the expected differences in model output.

Interestingly, the mean correlation of GNet8r and GNet8j model outputs was the largest, and quite high in absolute terms, even in V4. This means that the kind of optimization performed (i.e. task- vs. brain optimization) induced larger differences in model output than the presence or absence of hierarchical representation. It also means that the presence or absence of compositional or entailment hierarchy can be determined by very subtle differences in the output of a model.

## Discussion

We demonstrated that task- and brain-optimized networks can be made to yield highly accurate predictions of brain activity. We then investigated the kinds of hierarchical representation expressed by encoding models based on both single-branch (AlexNet and GNet8j) and multi-branch (GNet8r) DNNs. In encoding models based on AlexNet and GNet8j, lower layers contributed most strongly to posterior areas (evidence for compositional hierarchy), and representations optimized for anterior areas were more readily transferable to posterior areas than the other way around (evidence for entailment hierarchy). In contrast, for GNet8r the lower layers of each branch contributed equally to posterior and anterior areas, and optimized representations showed the same prediction accuracy shift when transferred to anterior or posterior areas. Thus, we conclude that compositional and entailment hierarchies are not necessary for accurate predictions of brain activity. Importantly, both GNet8j and GNet8r revealed an expansion of receptive field size (for fixed eccentricity) across V1–V4, indicating that, of the kinds of hierarchy we investigated, only integration hierarchy was formed by all model types.

We suspect that integration hierarchy is a special case of what we might call a “visual representation gradient”, i.e., a property of visual representations that changes monotonically with progression along a visual stream. Other well-known examples of representation gradients include the gradual reduction in spatial frequency preference^37^, increase in invariance^38^ and increase in “complexity”^39^ along the primate ventral stream. It is not known if any of these gradients are also examples of “hierarchies”, in any meaningful sense, or if they are a straightforward consequence of visual representations varying smoothly between pairs of points on the cortical sheet.

Integration hierarchy is sometimes discussed as if it is synonymous with the other kinds of hierarchy that, in our hands, had no essential value for predicting brain data. Our work thus underscores the importance of specifying the precise intended meaning of the word “hierarchy” when it is used to describe visual representation. The notion of hierarchy is complex and has many different possible meanings, each of which must be carefully assessed and tested against experimental and computational modeling results. We note that alternatives to composition hierarchy have been proposed to account for integration hierarchy^40^, again showing the independence of these concepts.

An intriguing aspect of our results is that both task- and brain-optimized single-branch models express all three kinds of hierarchy tested here. We interpret this as evidence that single-branch architectures enforce a correlation between integration and compositional hierarchies. Given the well-established increase in RF sizes across brain areas, it is not surprising that single-branch models learn to distribute representations encoded in higher brain areas to deep layers (in which each pixel has a “wider view” of the image than in layers below). This will necessarily lead to representations that form compositional and entailment hierarchies. By demonstrating the predictive performance of multi-branch models, we have shown the compositional and entailment hierarchies that form as a consequence of the vertical distribution of representations in single-branch models are not needed to accurately predict brain activity.

**Fig. 9:**
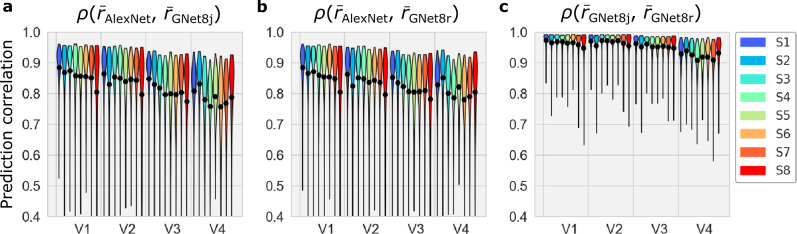
Correlation between the outputs of each pair of encoding models. **a**–**c** The distribution (violin plots; black dot indicates mean) of correlations (*ρ*) between the outputs ($\bar{r}$) of the indicated encoding models (subscript). Source data are provided as a Source Data file.

Our work indicates that the relationship between DNN architecture (i.e., the basic network connectivity diagram that specifies all of the connections between units in a network that can take on non-zero values) and representation (i.e., the set of stimulus features that are encoded in the activity of network units once the values of the connection weights are fixed) can be quite tenuous. The key findings that support this conclusion are that DNNs with very different architectures and objective functions yield nearly identical accuracy when read-out into predictions of brain activity (Fig. 4), and that models that vary in the kinds of hierarchy they express can have highly correlated outputs (Fig. 9).

Although we emphasize that the kinds of hierarchy we investigate in this work relate only to representations, not anatomy, our work suggests that the relationship between DNN architecture and cortical anatomy is also very tenuous. Visual cortex expresses several very clear kinds of hierarchy at the anatomical level. The classic hierarchy of Felleman and Van Essen^3^ is defined by the laminar distributions of axons connecting pairs of brain areas. The so-called “hodological” hierarchy^41^ is defined by the minimum number of synapses that interpose between the eye and a given brain area. A hierarchy of causal dependence can be inferred from inactivation studies^18–20^. Single-branch DNN architectures are superficially compatible with the latter kinds of anatomical hierarchy, whereas GNet8r is compatible with none. Given that models based on these different DNNs predict brain activity with the same level of accuracy, we can draw no anatomical lessons from the predictive success of either model. In particular, the GNet8r model, when interpreted as a model of anatomical connections, implies a lesson about anatomy that is obviously wrong: one could remove the V1 branch from the model without affecting the V2–V4 branches, whereas removing V1 from the brain would eliminate activity in V2–V4.

Other studies of DNN-based encoding models have also revealed qualitatively similar disconnects between DNN architecture and anatomy. For example, despite the fact that the visual cortex is full of recurrent connections, very deep but strictly feedforward DNNs can predict neural signals just as accurately as recurrent DNNs, even when care is taken to isolate neural signals that can be safely attributed to feedback^42^. Given their considerable flexibility and status as universal function approximators, DNNs can learn to emulate the outputs of systems whose components have no obvious mapping to the components of the DNN.

These findings should inform how we interpret the great success that DNNs have enjoyed as the foundation of SOTA encoding models in visual neuroscience. The architecture of single-branch DNNs (e.g., AlexNet) invites a mechanistic interpretation in which distinct brain areas are equated with layers, and in which the compositional and entailment hierarchies of the DNN itself are critical to the emergence of increasingly complex representations in these brain areas. Our results contribute to the ongoing debate about the validity of this mechanistic interpretation of DNNs^4,16,43–45^. The key to the success of the diverse DNN-based models in our work was clearly not any superficial resemblance between DNN architecture and cortical anatomy, nor was it the enforcement of hierarchical representations, since neither was needed for accurate prediction. This suggests that instead of crediting the architecture of DNNs with the success of DNN-based encoding models, it would be fruitful to investigate the outputs of the models themselves, treating them as approximations to brain activity that define a noiseless neural space whose functional and geometric properties can be probed for attributes that make models more or less accurate predictors of brain activity.

Our findings also underscore the need for researchers to clarify the specific kinds of hierarchy they are invoking when describing the visual system as “hierarchical”. The varieties of representational and anatomical hierarchy discussed here are not a full list of the hierarchies that have been (or could be) used to describe the primate visual system, and there is currently much uncertainty about how they all relate to one another^17,41^. We have shown that the three kinds of representational hierarchy considered here are distinct, but the same can be said for different kinds of anatomical hierarchy. For example, the hodological hierarchy is much more shallow than the classic hierarchy, and neither can fully account^41^ for the hierarchy of response onset latencies^46^ that themselves do not align with measured expansions of receptive field size (i.e., V3 has smaller onset latencies than V2, but larger receptive field sizes). Although they clearly require further refinement, DNNs are likely to serve as a helpful tool for understanding the relationship between the various kinds of representational and anatomical hierarchies, and for identifying which ones are essential for constructing intelligent visual systems.

Our work bears on a longstanding question of great importance to both neuroscience and AI: Why does the brain maintain a multitude of diverse representations at each point in visual space? The answer implied by the success of task-optimized DNNs is that the diversity of representations reflects incremental and progressive approximations toward an ideal that optimizes a single (or a few) behaviorally relevant objective(s). Hierarchies of successive approximations are necessary, under this view, because the ultimate representations have a highly nonlinear relationship to the stimulus, and must be constructed via the serial composition of relatively simple processing stages^16^. According to this perspective, the presence of diverse representations in the visual system reflects a processing constraint, and does not necessarily imply an equal diversity of functions.

The results in this work suggest that a very different interpretation of the function of diverse visual representations is equally plausible. The four branches of the GNet8j model encode representations that do not, collectively, imply serial processing. It is thus quite possible that, instead of forming a serial processing cascade that supports a single function, the diverse representations in V1–V4 may subserve diverse and independent functions that are distributed across the visual areas. The diverse nonlinearities required to support these putatively independent functions could in principle be implemented by (variable) local cortical processing, or by the input-output transformations performed by individual neurons, which have been shown to be implicitly “deep”^47–49^. If true, this would imply that for AI systems to embody brain-like visual representations, they will need to simultaneously satisfy multiple task objectives. We note that there is already considerable ongoing work along these lines^36,50,51^.

Testing this novel hypothesis about the diversity of representations across V1–V4 will require new analytical tools for uncovering the function of representations encoded in different human visual areas. The brain-optimized networks we introduce in this work will be helpful in this regard. Unlike task-optimized models, brain-optimized models learn brain-like representations while remaining agnostic to their function. Once learned, these representations can be tested for the ability to solve various tasks. Thus, rather than optimizing various networks against different task objectives and testing each network’s ability to predict brain activity, one could learn brain representations directly using brain-optimized networks, and compare the suitability of these representations for solving diverse tasks^52^.

We have used fMRI to test ideas about the relationship between DNNs and visual representation in the human brain. fMRI offers some compelling advantages over other signal modalities, but it also has obvious limitations that need to be considered when interpreting our results.

The primary advantage of fMRI is that it offers whole-brain coverage, thus providing access to a rich diversity of representations across multiple brain areas. This makes fMRI well-suited to studying how these distinct representations relate to one another.

A potential disadvantage of fMRI comes from its relatively poor temporal resolution. When analyzing fMRI data, we collapse the time-varying BOLD response to each image to a single number (the so-called “beta value”) that indicates the overall amplitude of the BOLD activity evoked by the image in a single voxel. Collapsing time-varying responses to a single activation value is a common practice in visual neuroscience^53,54^. For example, in electrophysiological studies of isolated single units, the average firing rate estimated from a fixed window that lags stimulus onset is taken as the effective activation amplitude of a neuron and is used to inform subsequent models of visual representation. However, given the relatively poor temporal resolution of fMRI, it is unavoidable that our estimates of per-image activation for each voxel intermix feedforward and feedback effects.

Although the function of feedback activity has long been debated^55^, it is possible it encodes representations that are less hierarchical than strictly feedforward activity. Even if this is the case, and even if fMRI beta values are relatively biased in favor of feedback effects, fMRI is still clearly a viable and important tool for understanding hierarchical representations in the human brain. fMRI data reveal a rich diversity of representations across V1–V4 that are remarkably well-aligned with representations revealed by analysis of single- and multi-unit activity^21,56^ Thus, fMRI is well-suited to address the question of whether the diverse representations in V1–V4 organize into a hierarchy. We have found that hierarchy does not seem to be an essential driver of the diversity of representations across V1–V4 (c.f., Fig. 9).

An important future direction for our work will be to apply our analyses to faster brain activity measures (e.g. spike rates) to determine if there are kinds of hierarchy that vary dynamically over time^57^. In particular, it will be interesting to determine if compositional and/or entailment hierarchies are required to predict brain activity at short latencies after stimulus presentation, when feedforward pathways make a dominant contribution to shaping the expected response to a stimulus. For example, one could expand the rows and columns of the prediction shift matrix (Fig. 7) to include distinct time points, and then determine if entailment hierarchy immediately after stimulus onset is an essential component of all accurate models. The concepts and analyses introduced here are thus likely to be very useful beyond the analysis of fMRI data, and could potentially provide insight into circuit function and dynamics.

## Methods

### Dataset acquisition

All data acquisition procedures were approved by the University of Minnesota Institutional Review Board.

All models were trained on the Natural Scenes Dataset (NSD^34^). The NSD dataset consists of between 22K and 30K fMRI image-responses per subject (8 subjects). Images were sampled from the Common Objects in Context (COCO) database^35^ and displayed at 8.4^∘^ × 8.4^∘^ (illustrative images in Figs. 2, 4 and 7 are property of the authors). The experimental design specified that each of the eight participants would view 10K distinct images (3 presentations each), and a special subset of 1K images would be shared across participants (8 subjects × 9K unique images + 1K shared images = 73K unique images); however, not all participants completed the full acquisition, so the final numbers are somewhat smaller. All fMRI data in the NSD were collected at ultra-high field (7T) using a whole-brain, 1.8-mm, 1.6-s, gradient-echo, echo-planar imaging (EPI) pulse sequence.

The image responses are expressed in terms of betas obtained from a general linear model (GLM) analysis. For this paper, we used GLM results provided with the NSD data release, specifically, the 1.8-mm volume preparation of the data and version 3 of the GLM betas (betas_fithrf_GLMdenoise_RR). This GLM version involves estimating the hemodynamic response function for each voxel, using the GLMdenoise technique for denoising^58^, and using ridge regression to improve the estimation of single-trial betas. Betas indicate BOLD response amplitudes evoked by each stimulus trial relative to the baseline signal level present during the absence of a stimulus ("gray screen”). The betas for each voxel in each session were separately z-scored and all sessions were concatenated.

### Experiment with artificial stimuli

In addition to the core NSD experiment, the 8 NSD subjects also participated in an additional 7T scanning session (these data will soon be released alongside the core NSD data). This session involved the presentation of a variety of artificial stimuli. Procedures for data acquisition, pre-processing, and GLM analysis were the same as for the NSD core. Stimuli consisted of a set of 284 images that can be conceptually grouped as follows (the number of distinct images in each group is indicated in parentheses): white noise (4), white noise with a large block size (4), pink noise (4), natural scenes (4), upside-down versions of these scenes (4), Mooney versions of these scenes (4), line-drawing versions of these scenes (4), contrast-modulated natural scenes (4 scenes × 5 contrast levels (100%, 50%, 10%, 6%, 4%) = 20), phase-coherence-modulated natural scenes (4 scenes × 4 coherence levels (75%, 50%, 25%, 0%) = 16), single words (4 words × 5 positions × 2 word lengths = 40), spiral gratings varying in orientation and spatial frequency (112), and chromatic pink noise varying in hue (68). Images typically occupied 8. 4^∘^ × 8. 4^∘^ (same as NSD core), though a few of the word stimuli extended beyond this extent.

Stimuli were presented in pseudorandom order using a 2-s ON/2-s OFF trial structure. Stimuli were shown against a gray background with an RGB value of (126, 110, 108), and were delivered using a linear color lookup table. During each run, a small semi-transparent gray fixation dot with a black border (0. 2^∘^ × 0. 2^∘^, 50% opacity) was present at the center of the stimuli. The luminance of the dot changed every 1.4 s. In alternating runs, while maintaining central fixation, subjects either performed a fixation task (report direction of the luminance change of the fixation dot) or a one-back task (report whether the current image is the same as the previous image). A total of 8 runs (each with duration 428 s) were collected, yielding a total of 744 stimulus trials over the course of the scan session. For the analyses performed in this paper, we modeled each stimulus trial (ignoring the variations in task performed by the subject) and considered only the central 8.4^∘^ square region (matching NSD core).

### Identification of visual brain areas V1–V4

Human visual brain areas were identified using a separate population receptive field (pRF) retinotopic mapping experiment, as documented in^34^. Retinotopic areas (more generally described as ‘regions of interest’ (ROIs)) were manually drawn based on results of the pRF experiment. These ROIs consist of V1v, V1d, V2v, V2d, V3v, V3d, and V4, and extend from the fovea (0^∘^ eccentricity) to peripheral locations that exhibit sensible responses in the pRF experiment given the limited stimulus size (the diameter of the pRF mapping stimulus was 8. 4^∘^). The total number of voxels (cumulative over subjects) in each ROI was 9041, 8818, 7763 and 3975 for V1, V2, V3 and V4 respectively, totaling 29597 voxels, with one subject contributing as few as 3027 voxels, and another as many as 4627 voxels.

### General encoding model architecture

Encoding models based upon both the task-optimized and brain-optimized networks consisted of a feature extractor (a DNN) and multiple read-out heads as detailed in the following sections and described in Allen et al.^34^.

#### Feature extractor

Feature extractors for all encoding models are sequences of transformations

1 $${e}_{L}(x)={\eta }_{L}\circ {e}_{L-1}(x)$$

operating on *x*, here an input image, where *η*_*L*_ is the transformation that operates at layer *L* on the output of the subsequence *e*_*L*−1_(*x*). *e*_*L*−1_(*x*) and *η*_*L*_ may themselves denote arbitrary sequences of transformations. Our encoding models leverage the multiple intermediate representations *e*_*l*_(*x*), which are feature maps whose elements are denoted by ${[{e}_{l}(x)]}_{kji}$, where *k* indexes features and (*i*, *j*) are pixel coordinates in each feature map.

#### Read-out heads

Read-out heads convert features output by the feature extractor into predictions of brain activity, ${\bar{r}}_{v}$, for each voxel *v*. These predictions can be expressed as a linearized model

2 $${\bar{r}}_{v}(x)={b}_{v}+\mathop{\sum}\limits_{k}{w}_{vk}\,{f}_{{{{{{\rm{out}}}}}}}({\Phi }_{k}(x))$$

where

3 $${\Phi }_{k}(x)=\mathop{\sum}\limits_{i,j}{f}_{{{{{{\rm{in}}}}}}}({[{e}_{1}(x)]}_{{k}_{1}\,ji}){g}_{vji}^{1}\oplus \cdots \mathop{\sum}\limits_{i,\,j}{f}_{{{{{{\rm{in}}}}}}}({[{e}_{L}(x)]}_{{k}_{L}\,ji}){g}_{vji}^{L}$$

and where *f*_in_( ⋅ ) and *f*_out_( ⋅ ) are typically some compressive nonlinearity and the sum ⊕ denotes the concatenation along the feature axis *k* = (*k*_1_, …*k*_*L*_). ${g}_{vji}^{l}$ is the value of the “pooling field” for voxel *v* applied at feature maps in layer *l* at pixel (*i*, *j*). Pooling fields generalize the population receptive field^21^ to arbitrary feature maps. All pooling field elements are positive-valued and normalized such that their sum equals to unity. Figure 2 illustrates the two pooling field variants used in our work. Gaussian pooling fields (gpf) are fully described by three parameters that specify the position and size of a symmetric 2D Gaussian function. For “flexible” pooling fields (fpf) each pixel value of the pooling field is an independent and learnable parameter.

### AlexNet encoding model

#### Feature extractor

For the task-optimized model featured in all figures of the main text, the feature extractor was an AlexNet deep convolutional neural network trained to classify 1000 object categories of the ImageNet database^7^. We used the pre-trained weights from Torchvision’s model zoo (https://pytorch.org/vision/stable/models.html). Not all feature maps were used in the encoding. At each layer specified in Supplementary Table 3, if a layer had more than 512 feature maps, we selected the 512 feature maps with the most variance with respect to the COCO images in our experiment. The final model thus exposed a total of 2688 feature maps.

#### Read-out head

We constructed two variants of the AlexNet model: one with a Gaussian pooling field (AlexNet-gpf), and one with a flexible pooling field (AlexNet-fpf). To construct AlexNet-fpf models, feature maps with the same spatial resolution were concatenated and a distinct spatial pooling field was learned for each spatial resolution. Thus, for AlexNet-fpf models the read-out heads included multiple pooling fields. For both model variants, feature maps from each layer throughout the depth of the AlexNet feature extractor were input to the read-out head. For details see Supplementary Table 3.

#### Training

For AlexNet models the parameters of the feature extractor were pre-trained, as described above. Thus, only the parameters of the read-out heads were optimized.

For AlexNet-gpf models the three pooling field parameters are learned via grid search over a list of 2680 candidates tiling the visual field with 8 log-spaced sizes varying from 3 to 40% of stimulus size and spaced roughly in proportion to their sizes (such that each size tiles the visual field fully). For each candidate receptive field, the tuning weights are learned via ridge regression with the ridge parameter selected to maximize validation accuracy on a held-out 10% set of the training set.

For AlexNet-fpf models the training of the read-out heads was performed via gradient descent with the ADAM optimizer (lr = 10^−3^, *β*_1_ = 0.9, *β*_2_ = 0.999).

### GNet encoding model

#### Feature extractor

We refer to the feature extractor of the brain-optimized network as a “GNet”. The GNet feature extractor consists of a pre-filtering network (*e*_1_(*x*)) followed by a deep feature extractor.

The input image resolution of the pre-filtering network is 227 × 227 and outputs an embedding of 192 features at a 27 × 27 spatial resolution. This part of the network is identical to the first two layers AlexNet.

The deep feature extractor is a sequence of blocks of layers. Each block consists of a batch normalization layer followed by a dropout layer and a convolutional layer that preserves feature map resolution. The use of batch normalization followed by dropout in each block has been characterized as performing input whitening and decorrelation^59^. After three blocks, the resolution of the feature maps is reduced from 25 × 25 to 13 × 13 by a max pooling layer. The GNet sequence of layers was designed so that each pixel in the final convolutional layer effectively pools over the whole input image, whereas pixels in lower layers only integrate over more local regions of the input image.

#### Read-out head

For the GNet model only flexible pooling fields were used due to training requirement. As with the AlexNet model, feature maps with the same spatial resolution were concatenated and a single spatial pooling field was learned for each spatial resolution. Thus, the read-out heads contained multiple pooling fields.

Read-out heads employed a fully differentiable nonlinearity:

4 $$f(x)=\tanh (x)\log (1+|x|),$$

that was applied either before or after spatial pooling, or both. This nonlinearity has several interesting and desirable characteristics: 1) it has an expansive and a compressive regime, 2) it is differentiable everywhere, with no discontinuity and 3) it does not plateau over a large range. The final GNet model benefited from using this nonlinearity before and after spatial pooling (i.e. as *f*_in_ and *f*_out_).

During the structure and hyperparameter selection process for GNet, we noticed that the best extant GNet model was obtained when some feature maps in lower layers were not directly connected to the read-out heads, as indicated on Supplementary Table 3. Note, however, we find that the results of analyses reported here are unchanged when using fully connected read-out heads.

#### Training

The pre-filtering network was taken from a task-optimized AlexNet. Its parameters were kept fixed during brain-optimization. While it was possible to optimize the pre-filtering network parameters along with the deep extractor parameters from a random initial condition, the results using a pre-trained filtering network were slightly better for all model variants.

All parameters of the deep feature extractor and the read-out heads are learned jointly via gradient descent with the ADAM optimizer (lr = 10^−3^, *β*_1_ = 0.9, *β*_2_ = 0.999). The training steps of the deep feature extractor and the read-out heads are alternated to promote stability of the training procedure.

To account for the noise profile and the discrepancies in voxel predictability, we used the following *L*2-norm weighted loss function

5 $$L(r,\bar{r}(x))=\frac{{\sum }_{v\in V}\lfloor {\rho }_{v}^{2}\rfloor {({r}_{v}-{\bar{r}}_{v}(x))}^{2}}{{\sum }_{v\in V}\lfloor {\rho }_{v}^{2}\rfloor },$$

where $\lfloor {\rho }_{v}^{2}\rfloor$ is the batchwise Pearson-like correlation of every voxel with a floor of 0.1 to always permit some contribution of the yet-to-be-predicted voxels (i.e. those with low correlation at the onset of training) in the voxel ensemble *V*. If the model can be trained voxelwise, then this weighting could be ignored but this could affect the learning dynamics (learning rates).

Unlike a typical deep network optimization with gradient descent, where all parameters subserve the global objective, this problem involves shared parameters (feature extractor) and voxelwise parameters (read-out heads). A global early stopping criterion may not be optimal for all voxels, since overfitting would be detected when the average loss starts to increase. On the other hand, voxelwise loss may increase before the optimal point due to the dependence on the changing deep feature extractor. This remains an outstanding problem. In spite of the aforementioned issues, relative successes were achieved by using a global early stopping criterion with the parameters clamped to their best value according to the validation accuracy of a 10% holdout set of the training set.

A second challenge emerged from the fact that each read-out head only has access to the brain responses associated with the subject-wise image samples, while the feature extractor leverages all available images. We addressed this issue by interleaving samples from each subject. While typical training consists of randomly sampling a minibatch from the training set, we first selected a subject at random and sampled a minibatch from its set of training images and brain responses. The read-out heads follow the matching brain response voxels and changes from batch to batch while the shared feature extractor is trained with gradients backpropagated from the current read-out heads. At the end of each epoch, every sample from every subject has been “seen” exactly once. This procedure yielded distinct improvements in model accuracy relative to subject-wise training, i.e. training a feature extractor network for each subject separately (as in^34^).

In some cases, we also performed a second and third phase of training (which we refer to as “fine tuning”, GNet8jft-fpf in Fig. 3e). Each subsequent phase restarts training from the optimal weight values from the previous phase. The second phase follows the same procedure as the first phase but all the read-out heads parameters were fixed and only the feature extractor parameters were trained (including those of the pre-filtering network). This second phase accounted for most of the improvement over the first phase. A third phase followed again the same objective in which we trained only the parameters of the read-out heads, while the feature extractor remained constant.

### Gabor encoding model

#### Feature extractor

The Gabor model feature extractor consists of a single fixed set of convolutions: 12 Gabor wavelets with spatial frequency log-spaced between 3 and 72 cyc/stimulus at 6 evenly-spaced orientations between 0 and *π*.

#### Read-out head

We used a read-out head with a Gaussian pooling field for the Gabor model. Following previous work^23^, we used a compressive nonlinearity ${f}_{{{{{{\rm{in}}}}}}}(x)=\log (1+|x|)$ while *f*_out_(*x*) = *x*.

#### Training

Gabor models were fit using grid search over the pooling field parameters (same candidate grid as for the AlexNet-gpf model), followed by ridge regression to determine the feature weights.

### Cross-validated prediction accuracy

Prediction accuracy is calculated using the subset of trials that include images displayed to all subjects (for most subjects, 1000 images with 3 repeats each). These trials were not included in the training data, and were not used for hyperparameter selection (see below), ensuring proper cross-validation. Prediction accuracy is the Pearson correlation between predicted brain activity and measured brain activity on a single-trial basis. Prediction accuracy uncertainty was estimated by sampling with replacement the predicted and actual brain activities for each voxel.

### Hyperparameter selection

The process of hyperparameter selection for the GNet models (e.g., pooling map resolutions, number of layers, nonlinearities, forms of regularization) was based on the prediction accuracy measured on a fixed, held-out model selection set consisting of 10% of each subject’s training data. The holdout prediction accuracy was evaluated, for each selection of hyperparameter values, at the early stopping point (i.e. at the minimum of the holdout loss during training of that model). The GNet model was refined on this basis using 4 subjects, and the best extant model was used to perform the final analysis with all 8 subjects.

### Receptive field modeling

Receptive fields (Fig. 5) were derived directly from the spatial pooling fields of the read-out head for each voxel. For some voxels, the flexible pooling fields do not have a clearly localized structure. However, for the vast majority of voxels the trained flexible spatial pooling fields extend smoothly from a clearly identifiable center. Thus, we characterized these maps by fitting an elliptical 2D gaussian to the parameters (the pixels in the map) of the flexible spatial pooling field. For each voxel, the size of its receptive field is defined as $\sqrt{\pi ab}$, where *a* and *b* are one std. dev. along the major and minor axis of the elliptical gaussian, and its eccentricity is the Euclidean distance from the display center. In the plot of Fig. 5d both measures are expressed as percent of the display size (i.e. 100% ≡ 8. 4^∘^).

### Layer-wise contributions to prediction accuracy

To test for alignment between layer depth and brain areas we calculated the prediction accuracy of network-based encoding models when layers from the bottom or top half of the feature extractor network were masked. Specifically, let *ρ*_bottom_ be the prediction accuracy obtained from an encoding model in which feature weights *w* from layers in the top half of the feature extractor network have been zeroed-out (refer to Fig. 6a for the designation of bottom and top layers in the AlexNet and GNet feature extractors, respectively). Separately, we also calculate the prediction accuracy *ρ*_top_ obtained by zeroing-out the feature weights that couple to the bottom layers. Both of these measures of prediction accuracy are necessarily smaller than the *total* prediction accuracy of the model *ρ* obtained when all feature weights are used (see Venn diagram in Fig. 6b).

To assess ordering for a given DNN, we calculated the specific and unique contributions of the bottom layers to predicting brain activity. Given that total variance varies a great deal across voxels, we express the specific and unique contributions of the bottom layers for each voxel in relative terms:

6 $${f}_{{{{{{\rm{bottom}}}}}}}^{{{{{{\rm{specific}}}}}}}=\frac{{\rho }_{{{{{{\rm{bottom}}}}}}}^{2}}{{\rho }^{2}}\quad \quad {{{{{\rm{and}}}}}}\quad \quad {f}_{{{{{{\rm{bottom}}}}}}}^{{{{{{\rm{unique}}}}}}}=\frac{{\rho }^{2}-{\rho }_{{{{{{\rm{top}}}}}}}^{2}}{{\rho }^{2}}$$

This decomposition of prediction accuracy into specific and unique contributions is performed for individual voxels. We then average the specific and unique contributions over all voxels in a single brain area that have validation accuracy that exceeded *ρ* = 0.055 (*p* < 0.01, prediction randomization trial). We plot these values along the presumed hierarchy of brain areas in Fig. 6c. We verified that the results were robust to the precise choice of threshold. Note that values for the specific and unique contribution of the top layer are obtained by swapping “bottom” and “top” subscripts in the above formulas, and can be read-off by eye from the curves in Fig. 6c.

### Transfer learning experiments

In transfer learning experiments, we test how well representations in a GNet feature extractor optimized for one brain area can generalize to another brain area. To test this, we first train a GNet feature extractor and read-out head to predict brain activity (or the outputs of another encoding model) for all voxels in a single brain area *i*. We then freeze the parameters of the trained GNet feature extractor for this area-specific encoding model (we call it the “reference model” below), but train new read-out heads to predict brain activity in all brain areas. We then compare the cross-validated prediction accuracy obtained when the feature extractor and read-out are trained on the same brain area (the reference model) to prediction accuracy obtained when the feature extractor and read-out are trained on different brain areas (the “transfer” model).

Let *i*, *j* ∈ (1, 2, 3, 4) index brain areas, and let ${\rho }_{v}^{{{{{{\rm{ref}}}}}}}$ be the cross-validated prediction accuracy of a reference model for voxel *v*. For the reference model, the GNet feature extractor and the read-out head are trained simultaneously on the brain area that *v* belongs to and then used to predict activity on held-out trials for that area. Let ${\rho }_{v}^{{V}_{j}}$ be the cross-validated prediction accuracy of a transfer model. For the transfer model, the feature extractor is trained on area *V*_*j*_ and then frozen while a read-out head is subsequently trained on the brain area to which *v* belongs. The transfer model is then used to predict activity on held-out trials in the brain area to which *v* belongs. We define the “prediction accuracy shift” ${\Delta }_{i}^{j}$ as:

7 $${\Delta }_{i}^{j}\equiv {\langle {\rho }_{v}^{{V}_{j}}-{\rho }_{v}^{{{{{{\rm{ref}}}}}}}\rangle }_{v\in {V}_{i}}$$

where 〈⋅〉_*v*∈*Vi*_ denotes averaging over all voxels in brain area *Vi*. In Figs. 7 and 8 we show prediction accuracy shift matrices consisting of ${\Delta }_{i}^{j}$ for all pairs of brain areas *V*_*i*_, *V*_*j*_. Rows (labeled “feature extractor trained on”) and columns (labeled “model tested on”) correspond to the superscript and subscript of ${\Delta }_{i}^{j}$, respectively. For entries along the diagonal of this matrix, ${\Delta }_{i}^{i}$ measures the prediction accuracy shift induced by first training the feature extractor and read-out head simultaneously on area *V*_*i*_ (i.e., the reference model) and then fixing the feature extractor and re-training the read-out head only on area *i* (i.e., the transfer model). The subtle difference between simultaneous and serial training of the model components typically induces a small positive prediction accuracy shift.

In Fig. 7 we illustrate the transfer of a GNet feature extractor optimized for V4 to V1,V2 and V3. The results of this transfer learning operation populate the top row, ${\Delta }_{i}^{4}$, of the prediction accuracy shift matrix.

In Fig. 8a, the three prediction accuracy shift matrices on the left labeled “AlexNet-gpf”, “GNet8j-fpf” and “GNet8r-fpf” were constructed by applying the transfer learning procedure just described to the outputs of the AlexNet-gpf, GNet8j-fpf and GNet8r-fpf models, respectively. In other words, instead of using brain activity to train the reference and transfer models, we use the outputs of the indicated encoding models to train them (by “outputs”, we specifically mean the activity predictions of the encoding model for each voxel). This allows us to identify the models that learn hierarchical representations during the course of their training. For the matrix on the far right labeled “Measured brain activity”, the transfer learning procedure was applied directly to measured brain activity. Note that in this special case, ${\rho }_{v}^{{{{{{\rm{ref}}}}}}}={\rho }_{v}^{{{{{{\rm{GNet8r}}}}}}}$ for all *v*.

We characterized the structure of the prediction accuracy shift matrices by calculating a scalar value, *α*, that captures the normalized difference between the upper and lower triangular components:

8 $$\alpha=\frac{{a}_{+}-{a}_{-}}{|{a}_{+}|+|{a}_{-}|}$$

where *a*_+_ and *a*_−_ are the slopes of the upper and lower triangular entries of the matrix, respectively. To calculate these slopes, we expressed the respective matrix entries as a function of their taxicab distance from the matrix diagonal, the off-diagonal entries having a distance of 1 and so on. In cases where the matrix is characterized by a salient lower triangular (negative slope) and a flat (zero slope) upper triangular, *α* would be close to 1. On the other hand, if the matrix is symmetric, *α* would be zero. The error in *α* is estimated via error propagation of the estimate of the errors on the slopes.

### Reporting summary

Further information on research design is available in the Nature Portfolio Reporting Summary linked to this article.

## Supplementary information

Supplementary Information

Peer Review File

Reporting Summary

## Data Availability

The NSD dataset is freely available at http://naturalscenesdataset.org. The data are hosted in the cloud, allowing researchers to exploit high-performance cloud computing to efficiently analyze the dataset. For a complete description see Allen et al.^34^. Source data are provided with this paper.

## Source data

Source data

## Code availability

We provide an archive of code used in this study at https://github.com/styvesg/nsd_gnet8x.
