# Supplementary material for: Brain-optimized deep neural network models of human visual areas learn non-hierarchical representations
Source: Nat Commun. 2023 Jun 7;14:3329. doi: 10.1038/s41467-023-38674-4 (PMC10247700; doi:10.1038/s41467-023-38674-4)
Supplement: Supplementary file 1 — Supplementary Information [file 41467_2023_38674_MOESM1_ESM.pdf]

Supplementary tables

Abbreviation	Description
Gabor-gpf	Gabor wavelet feature extractor with a single voxelwise Gaussian pooling field.
AlexNet-gpf	AlexNet feature extractor with a single voxelwise Gaussian pooling field.
AlexNet-fpf	AlexNet feature extractor with one flexible pooling field for each feature map resolution.
GNet8j-fpf	GNet feature extractor trained on all brain areas jointly (j) on 8 subjects, with one flexible pooling field for each feature map resolution.
GNet8jft-fpf	GNet8j-fpf model with fine tuning optimization procedure.
GNet8r-fpf	GNet feature extractor trained on each brain area separately, i.e. ROI-wise (r), on 8 subjects, with one flexible pooling field for each feature map resolution.

Supplementary Table 1: Table of models. A brief description of the various encoding models.

Symbol	Description
x	An image
r	Empirical voxel activity
$\bar{r}$	Voxel prediction
$\bar{r}_v$	Voxel prediction of voxel v
V	A population of voxels
$\bar{r}^{\text{model}}$	Voxel prediction of a specific model
ρ^{model}	Pearson correlation between prediction of model and target activity (context dependent)
$e_l(x)$	l-th set of feature map of x
$[e_l(x)]_{kji}$	A specific feature of feature map $e_l(x)$
$\Phi(x)$	Spatially pooled feature of x
g_v^n	n-th pooling field of voxel v
w_v	Feature weight for voxel v
$\langle\cdot\rangle_V$	Average over voxel population V

Supplementary Table 2: Table of symbols Description of the most frequently used symbols.

Resolution	Select	Alexnet	GNet	Select	Resolution
		input: 227x227 color images	input: 227x227 color images		
		64 conv 11x11, str 4, pad 2	64 conv 11x11, str 4, pad 2		
(27x27)	64	maxpool 3x3, str 2, pad 0	maxpool 3x3, str 2, pad 0		
(27x27)	192	192 conv 5x5, str 1, pad 2	192 conv 5x5, str 1, pad 2		
		maxpool 3x3, str 2, pad 0	128 conv 3x3, str 1, pad 0	128	(25x25)
(13x13)	384	384 conv 3x3, str 1, pad 1	batchnorm + dropout		
(13x13)	256	256 conv 3x3, str 1, pad 1	128 conv 3x3, str 1, pad 1	64	(25x25)
(13x13)	256	256 conv 3x3, str 1, pad 1	batchnorm + dropout		
		maxpool 3x3, str 2, pad 0	128 conv 3x3, str 1, pad 1	64	(25x25)
		adaptive avg pool 6x6	maxpool 3x3, str 2, pad 1		
(1x1)	512	4096 fully con.	batchnorm + dropout		
(1x1)	512	4096 fully con.	128 conv 3x3, str 1, pad 1	64	(13x13)
(1x1)	512	1000 fully con.	batchnorm + dropout		
			128 conv 3x3, str 1, pad 1	64	(13x13)
			batchnorm + dropout		
			128 conv 3x3, str 1, pad 1	64	(13x13)
			batchnorm + dropout		
			64 conv 3x3, str 1, pad 1	64	(13x13)

Supplementary Table 3: Details of the feature extractor networks. Layer-by-layer network structure comparison between AlexNet and GNet. The number of feature maps connected to the read-out heads is indicated under the 'select' label. In the case of AlexNet, this selection is based on feature map variance w.r.t the NSD training set, whereas the GNet selection is based on a fixed partition of the feature maps at onset of training.

Supplementary figures

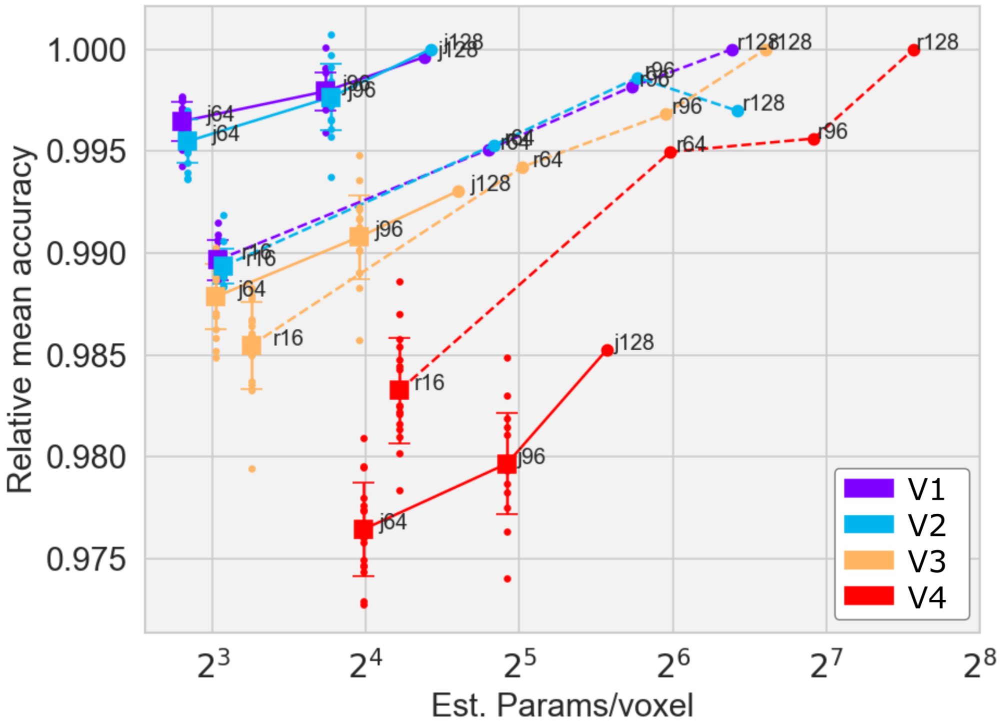

Supplementary Figure 1: The effect of model capacity on prediction accuracy of the jointly-trained, single-branch GNet8j and the roi-wise, multi-branch GNet8r. For each brain area V1–V4 we calculated mean prediction accuracy (y-axis) relative to the best-predicting model (solid lines, points labeled "j" indicate GNet8j; dashed lines, points labeled "r" indicate GNet8r) over a range of parameters-per-voxel (we considered only trainable parameters of the DNN feature extractor). Multiple random splits of the training/holdout data were performed for the three smallest models in order to estimate the standard deviation (error bars) of the mean. In no brain area does the best-performing single-branch model outperform the best multi-branch model. In V1 and V2, single-branch models outperformed multi-branch models when matched for capacity. In V3, capacity-matched single- and multi-branch models have close to identical prediction accuracy. In V4, multi-branch models outperform capacity-matched single-branch models. In all cases, variation in prediction accuracy was small (0.5 - 2%).
