# Supplementary material for: Brain-optimized deep neural network models of human visual areas learn non-hierarchical representations
Source: Nat Commun. 2023 Jun 7;14:3329. doi: 10.1038/s41467-023-38674-4 (PMC10247700; doi:10.1038/s41467-023-38674-4)
Supplement: Supplementary file 3 — Reporting Summary [file 41467_2023_38674_MOESM3_ESM.pdf]

nature portfolio

Corresponding author(s): Thomas Naselaris

Last updated by author(s): Mar 11, 2023

Reporting Summary

Nature Portfolio wishes to improve the reproducibility of the work that we publish. This form provides structure for consistency and transparency in reporting. For further information on Nature Portfolio policies, see our Editorial Policies and the Editorial Policy Checklist.

Statistics

For all statistical analyses, confirm that the following items are present in the figure legend, table legend, main text, or Methods section.

n/a	Confirmed	
☐	☒	The exact sample size (*n*) for each experimental group/condition, given as a discrete number and unit of measurement
☐	☒	A statement on whether measurements were taken from distinct samples or whether the same sample was measured repeatedly
☐	☒	The statistical test(s) used AND whether they are one- or two-sided<br>*Only common tests should be described solely by name; describe more complex techniques in the Methods section.*
☐	☒	A description of all covariates tested
☐	☒	A description of any assumptions or corrections, such as tests of normality and adjustment for multiple comparisons
☐	☒	A full description of the statistical parameters including central tendency (e.g. means) or other basic estimates (e.g. regression coefficient) AND variation (e.g. standard deviation) or associated estimates of uncertainty (e.g. confidence intervals)
☐	☒	For null hypothesis testing, the test statistic (e.g. *F*, *t*, *r*) with confidence intervals, effect sizes, degrees of freedom and *P* value noted<br>*Give P values as exact values whenever suitable.*
☒	☐	For Bayesian analysis, information on the choice of priors and Markov chain Monte Carlo settings
☒	☐	For hierarchical and complex designs, identification of the appropriate level for tests and full reporting of outcomes
☐	☒	Estimates of effect sizes (e.g. Cohen's *d*, Pearson's *r*), indicating how they were calculated

Our web collection on statistics for biologists contains articles on many of the points above.

Software and code

Policy information about availability of computer code

Data collection	Psychophysics Toolbox 3.0.14, MATLAB R2018a, Meadows web-based platform (http://meadows-research.com)
Data analysis	Data analysis was performed using python 3.6.8, numpy 1.19.5, scipy 1.5.4, torch 1.10 with CUDA 11.3 and cudnn 8.2

For manuscripts utilizing custom algorithms or software that are central to the research but not yet described in published literature, software must be made available to editors and reviewers. We strongly encourage code deposition in a community repository (e.g. GitHub). See the Nature Portfolio guidelines for submitting code & software for further information.

Data

Policy information about availability of data

All manuscripts must include a data availability statement. This statement should provide the following information, where applicable:

- Accession codes, unique identifiers, or web links for publicly available datasets
- A description of any restrictions on data availability
- For clinical datasets or third party data, please ensure that the statement adheres to our policy

The fMRI data used here come from the Natural Scenes Dataset (NSD). The NSD is freely available at http://naturalscenesdataset.org. The data are hosted in the cloud, allowing researchers to exploit high-performance cloud computing to efficiently analyze the dataset. We provide both raw data in BIDS format and prepared data files, along with extensive technical documentation in the NSD Data Manual. Images used for the NSD were taken from the Common Objects in Context database (https://cocodataset.org).

Human research participants

Policy information about studies involving human research participants and Sex and Gender in Research.

Reporting on sex and gender	A mixture of males and females were used (2m, 6f).
Population characteristics	All participants were healthy young adults between 19–32 years old, and all provided informed written consent. Participants were compensated at a rate of $30 per hour, plus performance bonuses.
Recruitment	Participants were recruited through advertisements to the local community and were screened based on ability to participate in this long-term neuroimaging study. In addition, we selected participants based on data quality from an initial 7T fMRI session. This selection does induce a bias towards individuals with low head motion, high cognitive performance, and strong BOLD responses. The goal of the NSD dataset is largely to create a massive dataset to inform studies of the basic mechanisms of vision and memory, and does not represent an unbiased sampling of the human population.
Ethics oversight	University of Minnesota Institutional Review Board

Note that full information on the approval of the study protocol must also be provided in the manuscript.

Field-specific reporting

Please select the one below that is the best fit for your research. If you are not sure, read the appropriate sections before making your selection.

☒ Life sciences ☐ Behavioural & social sciences ☐ Ecological, evolutionary & environmental sciences

For a reference copy of the document with all sections, see nature.com/documents/nr-reporting-summary-flat.pdf

Life sciences study design

All studies must disclose on these points even when the disclosure is negative.

Sample size	The NSD includes massive amounts of data in individual subjects. Analyses demonstrated in this paper are conducted primarily at the within-subject level, demonstrating the precision and robustness of the data collected. For group-level analyses, the number of subjects used for NSD (n = 8) is sufficiently large to provide some power for statistical inference.
Data exclusions	8 subjects out of a pool of 14 potential subjects (on basis of criteria such as head motion and BOLD signal strength) were selected for full NSD data acquisition.
Replication	All data analyses were applied to data from all 8 subjects in the NSD.
Randomization	All participants engaged in the same set of experiments. However, somewhat non-overlapping sets of stimuli were chosen for each subject. The allocation of stimuli to different subjects was done randomly from a fixed set of images pulled from the Microsoft COCO database. Given the large scale of stimulus sampling (e.g. 9,000–10,000 unique images were shown to each subject), it is likely that although the exact same images are not shown to each subject, the same general types of stimulus features are well sampled for each subject.
Blinding	Blinding is not relevant to this study given that there is little that the investigators could have done to bias the nature of the recorded data and given that the participants do not belong to any discrete groupings.

Reporting for specific materials, systems and methods

We require information from authors about some types of materials, experimental systems and methods used in many studies. Here, indicate whether each material, system or method listed is relevant to your study. If you are not sure if a list item applies to your research, read the appropriate section before selecting a response.

Materials & experimental systems

n/a	Involved in the study	
☒	☐	Antibodies
☒	☐	Eukaryotic cell lines
☒	☐	Palaeontology and archaeology
☐	☒	Animals and other organisms
☒	☐	Clinical data
☒	☐	Dual use research of concern

Methods

n/a	Involved in the study	
☒	☐	ChIP-seq
☒	☐	Flow cytometry
☐	☒	MRI-based neuroimaging

Animals and other research organisms

Policy information about studies involving animals; ARRIVE guidelines recommended for reporting animal research, and Sex and Gender in Research

Laboratory animals	n/a
Wild animals	n/a
Reporting on sex	n/a
Field-collected samples	n/a
Ethics oversight	n/a

Note that full information on the approval of the study protocol must also be provided in the manuscript.

Magnetic resonance imaging

Experimental design

Design type	The core NSD experiment is task-based and has an event-related design. The prf experiment is task-based and has a continuous design.
Design specifications	In the core NSD experiment, images were presented for 3 seconds, and were followed by a minimum of 1 second of gap before the next trial. Many thousands of distinct images were presented over the course of many distinct scan sessions, with a maximum number of presentations per distinct image of 3.
Behavioral performance measures	Button presses and associated reaction times for each trial in the NSD experiment were recorded. To ensure high data quality, we monitored basic response metrics, like response rate. We quantified recognition performance in the NSD experiment using signal detection theory.

Acquisition

Imaging type(s)	Functional, structural
Field strength	7T
Sequence & imaging parameters	The primary fMRI sequence involved gradient-echo EPI, FOV 216 mm x 216 mm, matrix size 120 x 120, slice thickness 1.8 mm, orientation axial, TR 1.6 s, TE 22.0 ms, and flip angle 62°.
Area of acquisition	Whole-brain scans including cerebellum

Diffusion MRI ☐ Used ☒ Not used

Preprocessing

Preprocessing software	A combination of custom MATLAB and Python code, FreeSurfer 6, and selected tools from SPM, FSL, ANTs, and MRTrix3.
Normalization	All analyses were conducted on data in subject-native spaces.
Normalization template	n/a
Noise and artifact removal	For the GLM preparation of the NSD data, the data-driven analysis method GLMdenoise and the statistical technique of ridge regression were used. These methods can account for a variety of sources of noise (e.g., physiological, motion, scanner artifacts, effects of collinearity). A version of the GLM results that omit these noise removal methods is also provided.
Volume censoring	n/a

Statistical modeling & inference

Model type and settings	Trial-wise fMRI response amplitudes were estimated for individual voxels in individual subjects.
Effect(s) tested	The effect of network architecture on the prediction accuracy of neural-network based encoding models is tested.

Specify type of analysis: ☐ Whole brain ☒ ROI-based ☐ Both

Anatomical location(s): V1, V2, V3, V4

Statistic type for inference (See Eklund et al. 2016)	Multiple multivariate statistics are used for inference.

Correction	n/a

Models & analysis

n/a	Involved in the study
☒	☐ Functional and/or effective connectivity
☒	☐ Graph analysis
☐	☒ Multivariate modeling or predictive analysis

Multivariate modeling and predictive analysis	For neural network modeling, we used either pre-trained image-computable neural network models (AlexNet, Gabor model) or brain-optimized image-computable neural network models (GNet). These models were trained on a set of training data (the non-shared NSD images) and validated on a separate set of validation data (the shared NSD images).
